# [4′-(4-Amino­phen­yl)-2,2′:6′,2′′-terpyridine]­chloridopalladium(II) chloride

**DOI:** 10.1107/S1600536811018125

**Published:** 2011-05-20

**Authors:** John C. Thomas, Edward R. T. Tiekink, Judith A. Walmsley

**Affiliations:** a Department of Chemistry, The University of Texas at San Antonio, One UTSA Circle, San Antonio, Texas 78249, USA; b Department of Chemistry, University of Malaya, 50603 Kuala Lumpur, Malaysia

## Abstract

The Pd^II^ atom in the complex cation of the title compound, [PdCl(C_21_H_16_N_4_)]Cl, is coordinated by three N atoms derived from the terpyridine ligand and a chloride ion, which define a distorted PdClN_3_ square-planar coordination geometry. In the crystal, the presence of N—H⋯Cl hydrogen bonds involving the amino H atom and chloride anions link two cations and two anions into a four-ion aggregate *via* centrosymmetric eight-membered [⋯H—N—H⋯Cl]_2_ synthons. Layers of cations are inter­spersed with the chloride anions with stabilization provided by C—H⋯Cl inter­actions involving both Cl atoms, as well as π–π inter­actions [the closest inter­action of 3.489 (6) Å occurs between a chelate ring and a pyridyl residue].

## Related literature

For background to metal–terpyridine complexes, see: Storrier *et al.* (1997 ▶); Hofmeier & Schubert (2004 ▶); Eryazici *et al.* (2008 ▶). For the synthesis, see: Tu *et al.* (2007 ▶); Laine *et al.* (2002 ▶).
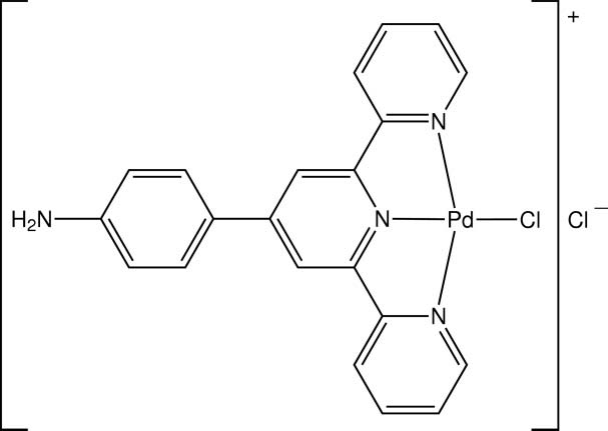


## Experimental

### 

#### Crystal data

[PdCl(C_21_H_16_N_4_)]Cl
*M* *_r_* = 501.68
Monoclinic, 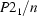

*a* = 10.853 (3) Å
*b* = 13.151 (3) Å
*c* = 13.654 (3) Å
β = 105.215 (6)°
*V* = 1880.5 (7) Å^3^
*Z* = 4
Mo *K*α radiation
μ = 1.29 mm^−1^
*T* = 98 K
0.10 × 0.04 × 0.04 mm

#### Data collection

Rigaku AFC12/SATURN724 diffractometer
Absorption correction: multi-scan (*ABSCOR*; Higashi, 1995 ▶) *T*_min_ = 0.412, *T*_max_ = 1
15599 measured reflections
3296 independent reflections
2809 reflections with *I* > 2σ(*I*)
*R*_int_ = 0.079

#### Refinement

*R*[*F* ^2^ > 2σ(*F* ^2^)] = 0.098
*wR*(*F* ^2^) = 0.239
*S* = 1.16
3296 reflections
259 parameters
3 restraints
H atoms treated by a mixture of independent and constrained refinement
Δρ_max_ = 3.59 e Å^−3^
Δρ_min_ = −2.15 e Å^−3^

### 

Data collection: *CrystalClear* (Molecular Structure Corporation & Rigaku, 2005 ▶); cell refinement: *CrystalClear*; data reduction: *CrystalClear*; program(s) used to solve structure: *PATTY* in *DIRDIF* (Beurskens *et al.*, 1992 ▶); program(s) used to refine structure: *SHELXL97* (Sheldrick, 2008 ▶); molecular graphics: *ORTEP-3* (Farrugia, 1997 ▶) and *DIAMOND* (Brandenburg, 2006 ▶); software used to prepare material for publication: *publCIF* (Westrip, 2010 ▶).

## Supplementary Material

Crystal structure: contains datablocks global, I. DOI: 10.1107/S1600536811018125/hb5880sup1.cif

Structure factors: contains datablocks I. DOI: 10.1107/S1600536811018125/hb5880Isup2.hkl

Additional supplementary materials:  crystallographic information; 3D view; checkCIF report

## Figures and Tables

**Table 1:** Selected bond lengths (Å)

Pd—N1	2.034 (10)
Pd—N2	1.922 (9)
Pd—N3	2.022 (10)
Pd—Cl1	2.290 (3)

**Table 2:** Hydrogen-bond geometry (Å, °)

*D*—H⋯*A*	*D*—H	H⋯*A*	*D*⋯*A*	*D*—H⋯*A*
N4—H1n⋯Cl2	0.88 (9)	2.42 (7)	3.287 (11)	167 (8)
N4—H2n⋯Cl2^i^	0.88 (7)	2.48 (8)	3.352 (10)	173 (10)
C13—H13⋯Cl1^ii^	0.95	2.61	3.493 (14)	156
C15—H15⋯Cl2^iii^	0.95	2.56	3.449 (12)	155

Symmetry codes: (i) 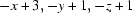; (ii) 
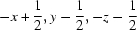
; (iii) 
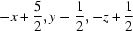
.

**Fig. 1.**
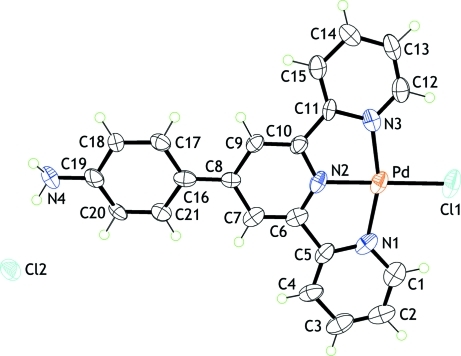
Molecular structure of (I) showing displacement ellipsoids at the 50% probability level.

**Fig. 2.**
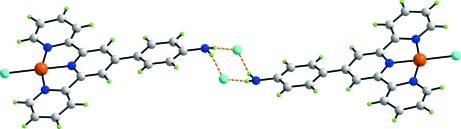
Four-ion aggregate in (I) mediated by N—H···Cl (orange dashed lines) hydrogen bonds.

**Fig. 3.**
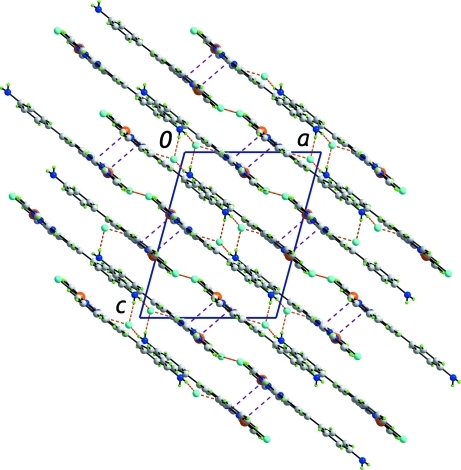
A view in projection down the b axis of the unit-cell contents for (I). The N—H···Cl, C—H···Cl and π–π interactions are shown as orange, blue and purple dashed lines, respectively.

## supplementary crystallographic information

### Comment

Interest in transition metal terpyridine complexes (Hofmeier & Schubert, 2004; Eryazici *et al.*, 2008) led to the synthesis and characterization of the title Pd^II^ complex, (I), featuring a rare example of a 4'-(4-anilino)-2,2':6',2''-terpyridine ligand (Storrier *et al.*, 1997).

Complex (I) comprises a [Pd{4'-(4-anilino)-2,2':6',2''-terpyridine}Cl] cation and a chloride anion, Fig. 1. The Pd^II^ center is coordinated by three N atoms derived from the tridentate terpyridine ligand, and the square planar geometry is completed by a Cl atom, Table 1. The Pd—N2 bond distance is significantly shorter than the remaining Pd—N bonds, an observation correlated with the restricted bite angles of the ligand (N1—Pd—N3 = 161.6 (4) °). Each of the five-membered chelate rings is essentially planar (r.m.s. = 0.021 and 0.028 Å) and the dihedral angle formed between these rings = 1.7 (4) °. With reference to the central N2-containing pyridyl ring, the N1- and N3-pyridyl rings form dihedral angles of 4.0 (6) and 6.0 (5) °, respectively; the dihedral angle between the wing pyridyl rings = 6.7 (6) °. A slight twist is found around the C8—C16 bond as seen in the C7—C8—C16—C17 torsion angle of 171.5 (9)°; the dihedral angle formed between these rings is 8.6 (5) °.

The most prominent feature of the crystal packing is the formation of N—H···Cl hydrogen bonds, Table 2, whereby cations are connected *via* centrosymmetric eight-membered [···H—N—H···Cl]_2_ synthons, Fig. 2. Globally, the cations aggregate into layers interspersed by the Cl anions. The are significant intralayer C—H···Cl contacts involving the covalently bound Cl as well as interlayer C—H···Cl^-^ contacts, Table 2. This pattern brings into proximity several π-systems with the closest π–π contact of 3.489 (6) Å occurring between the Pd,N1,N2,*C*_2_ chelate ring and a symmetry related N3-pyridyl ring [angle of inclination = 4.1 (2) ° for symmetry operation 1 - *x*, 1 - *y*, -*z*]. A view of the unit-cell contents is shown in Fig. 3.

### Experimental

Synthesis of 4'-(4-nitrophenyl)-2,2':6',2''-terpyridine, the precursor used for the synthesis of 4'-(4-anilino)-2,2':6',2''-terpyridine (*L*^1^). 4-Nitrobenzaldehyde (0.302 g, 2.00 mmol), 2-acetylpyridine (0.450 ml, 4.00 mmol), and ammonium acetate (0.308 g, 4.00 mmol) were mixed in a 10 ml reaction vial, suitable for microwave irradiation. Deionized H_2_O (2.00 ml) was added using a syringe to the reaction vial. The reaction vial was capped, and the mixture was irradiated *via* microwave at 200 W, 200 PSI, and at 403 K for 22 minutes with stirring (Tu *et al.*, 2007). The crude product was filtered, and dissolved in 100 ml 1-propanol. This solution was filtered *via* gravity filtration, concentrated and crystallized. Product was recrystallized in a 95% ethanol solution to afford a brown precipitate (0.098 g, 27.7%); *M*.pt. 434–435 K.

Synthesis of *L*^1^: 4'-4-nitrophenyl)-2,2':6',2''-terpyridine (0.098 g, 0.277 mmol) and Pd—C 10% (0.030 g) in 20 ml e thanol were refluxed for 1 h under nitrogen. Hydrazine hydrate solution (N_2_H_4_ 55%, 0.320 ml, 5.80 mmol, 21 equiv.) was added drop wise using a syringe. Reflux was carried out for an additional 30 minutes. The reaction mixture was cooled to room temperature and gravity filtered over cotton-wool. The catalyst was washed with CH_2_Cl_2_ (100 ml). The organic filtrates were washed with deionized H_2_O until neutrality was reached. The organic phase was dried over magnesium sulfate and filtered. The solvent was evaporated under heat, to yield a light brown microcrystalline product (Laine *et al.*, 2002). This was recrystallized from 95% ethanol to afford a yellow microcrystalline product (0.035 g, 36.7%); *M*.pt. 507–511 K.

Synthesis of [Pd{4'-(4-anilino)-2,2':6',2''-terpyridine}Cl]Cl: *L*^1^ (11.5 mg, 0.034 mmol) was dissolved in 10 ml me thanol and added to a solution of K_2_PdCl_4_ (11.0 mg, 0.034 mmol) in 10 ml me thanol in a 100 ml beaker. The solution was heated gently with stirring for 30 minutes and evaporated to afford a dark red crystalline product (9.80 mg, 53.3%). Red prisms of (I) were obtained by recrystallization from ethanol solution by vapor diffusion of diethylether.

### Refinement

C-bound H-atoms were placed in calculated positions (C—H = 0.95 Å) and were included in the refinement in the riding model approximation with *U*_iso_(H) set to 1.2*U*_eq_(C). The N-bound H-atoms were located in a difference Fourier map and refined with an N—H restraint of 0.880±0.001 Å, a H···H restraint of 1.43±0.01 Å, and with *U*_iso_(H) = 1.2*U*_eq_(N). The maximum and minimum residual electron density peaks of 3.59 and 2.15 e Å^-3^, respectively, were located 0.93 Å and 0.80 Å from the Pd atom.

### Crystal data

**Table:**

[PdCl(C_21_H_16_N_4_)]Cl	*F*(000) = 1000
*M**_r_* = 501.68	*D*_x_ = 1.772 Mg m^−^^3^
Monoclinic, *P*2_1_/*n*	Mo *K*α radiation, λ = 0.71070 Å
Hall symbol: -P 2yn	Cell parameters from 7657 reflections
*a* = 10.853 (3) Å	θ = 2.1–30.3°
*b* = 13.151 (3) Å	µ = 1.29 mm^−^^1^
*c* = 13.654 (3) Å	*T* = 98 K
β = 105.215 (6)°	Prism, red
*V* = 1880.5 (7) Å^3^	0.10 × 0.04 × 0.04 mm
*Z* = 4	

### Data collection

**Table:**

Rigaku AFC12/SATURN724 diffractometer	3296 independent reflections
Radiation source: fine-focus sealed tube	2809 reflections with *I* > 2σ(*I*)
graphite	*R*_int_ = 0.079
ω scans	θ_max_ = 25.0°, θ_min_ = 2.1°
Absorption correction: multi-scan (*ABSCOR*; Higashi, 1995)	*h* = −12→11
*T*_min_ = 0.412, *T*_max_ = 1	*k* = −15→15
15599 measured reflections	*l* = −16→16

### Refinement

**Table:**

Refinement on *F*^2^	Primary atom site location: structure-invariant direct methods
Least-squares matrix: full	Secondary atom site location: difference Fourier map
*R*[*F*^2^ > 2σ(*F*^2^)] = 0.098	Hydrogen site location: inferred from neighbouring sites
*wR*(*F*^2^) = 0.239	H atoms treated by a mixture of independent and constrained refinement
*S* = 1.16	*w* = 1/[σ^2^(*F*_o_^2^) + (0.094*P*)^2^ + 20.7984*P*] where *P* = (*F*_o_^2^ + 2*F*_c_^2^)/3
3296 reflections	(Δ/σ)_max_ < 0.001
259 parameters	Δρ_max_ = 3.59 e Å^−^^3^
3 restraints	Δρ_min_ = −2.15 e Å^−^^3^

### Special details

**Table:**

Geometry. All e.s.d.'s (except the e.s.d. in the dihedral angle between two l.s. planes) are estimated using the full covariance matrix. The cell e.s.d.'s are taken into account individually in the estimation of e.s.d.'s in distances, angles and torsion angles; correlations between e.s.d.'s in cell parameters are only used when they are defined by crystal symmetry. An approximate (isotropic) treatment of cell e.s.d.'s is used for estimating e.s.d.'s involving l.s. planes.
Refinement. Refinement of *F*^2^ against ALL reflections. The weighted *R*-factor *wR* and goodness of fit *S* are based on *F*^2^, conventional *R*-factors *R* are based on *F*, with *F* set to zero for negative *F*^2^. The threshold expression of *F*^2^ > σ(*F*^2^) is used only for calculating *R*-factors(gt) *etc*. and is not relevant to the choice of reflections for refinement. *R*-factors based on *F*^2^ are statistically about twice as large as those based on *F*, and *R*- factors based on ALL data will be even larger.

### Fractional atomic coordinates and isotropic or equivalent isotropic displacement parameters (Å^2^)

**Table:**

	*x*	*y*	*z*	*U*_iso_*/*U*_eq_	
Pd	0.52238 (8)	0.61084 (7)	−0.12448 (6)	0.0406 (4)	
Cl1	0.3256 (3)	0.6393 (3)	−0.2332 (2)	0.0602 (9)	
Cl2	1.5657 (3)	0.6930 (2)	0.4574 (2)	0.0521 (8)	
N1	0.5776 (9)	0.7570 (7)	−0.0888 (7)	0.042 (2)	
N2	0.6866 (8)	0.5872 (7)	−0.0316 (6)	0.037 (2)	
N3	0.5215 (8)	0.4571 (7)	−0.1273 (6)	0.038 (2)	
N4	1.4174 (9)	0.4831 (8)	0.3621 (7)	0.044 (2)	
H1N	1.469 (9)	0.534 (5)	0.387 (7)	0.053*	
H2N	1.428 (11)	0.435 (5)	0.408 (6)	0.053*	
C1	0.5076 (12)	0.8421 (10)	−0.1202 (9)	0.051 (3)	
H1	0.4249	0.8365	−0.1656	0.061*	
C2	0.5559 (14)	0.9367 (9)	−0.0865 (10)	0.053 (3)	
H2	0.5049	0.9954	−0.1074	0.063*	
C3	0.6765 (15)	0.9472 (10)	−0.0233 (10)	0.057 (4)	
H3	0.7108	1.0125	−0.0021	0.068*	
C4	0.7472 (13)	0.8592 (8)	0.0090 (9)	0.047 (3)	
H4	0.8303	0.8637	0.0539	0.056*	
C5	0.6961 (11)	0.7660 (8)	−0.0245 (8)	0.040 (3)	
C6	0.7595 (11)	0.6664 (8)	0.0078 (8)	0.037 (2)	
C7	0.8789 (11)	0.6510 (8)	0.0711 (8)	0.038 (3)	
H7	0.9300	0.7078	0.0993	0.045*	
C8	0.9263 (10)	0.5522 (7)	0.0949 (8)	0.033 (2)	
C9	0.8437 (10)	0.4710 (8)	0.0530 (7)	0.032 (2)	
H9	0.8708	0.4029	0.0690	0.038*	
C10	0.7247 (10)	0.4895 (8)	−0.0106 (8)	0.035 (2)	
C11	0.6283 (9)	0.4156 (8)	−0.0622 (8)	0.033 (2)	
C12	0.4354 (10)	0.3957 (10)	−0.1824 (8)	0.043 (3)	
H12	0.3622	0.4250	−0.2275	0.052*	
C13	0.4449 (11)	0.2911 (11)	−0.1785 (9)	0.051 (3)	
H13	0.3806	0.2501	−0.2211	0.061*	
C14	0.5489 (11)	0.2470 (9)	−0.1120 (8)	0.043 (3)	
H14	0.5565	0.1751	−0.1065	0.052*	
C15	0.6434 (10)	0.3105 (9)	−0.0527 (8)	0.038 (3)	
H15	0.7166	0.2822	−0.0067	0.046*	
C16	1.0522 (11)	0.5334 (8)	0.1615 (8)	0.036 (2)	
C17	1.0953 (11)	0.4342 (8)	0.1970 (8)	0.037 (2)	
H17	1.0409	0.3775	0.1750	0.045*	
C18	1.2149 (10)	0.4190 (8)	0.2630 (8)	0.036 (2)	
H18	1.2403	0.3521	0.2860	0.044*	
C19	1.2991 (10)	0.5001 (9)	0.2966 (8)	0.038 (3)	
C20	1.2611 (10)	0.5949 (8)	0.2590 (8)	0.033 (2)	
H20	1.3183	0.6504	0.2784	0.040*	
C21	1.1426 (10)	0.6120 (8)	0.1938 (8)	0.035 (2)	
H21	1.1208	0.6791	0.1697	0.042*	

### Atomic displacement parameters (Å^2^)

**Table:**

	*U*^11^	*U*^22^	*U*^33^	*U*^12^	*U*^13^	*U*^23^
Pd	0.0386 (6)	0.0509 (6)	0.0318 (5)	0.0157 (4)	0.0086 (4)	0.0086 (4)
Cl1	0.0460 (18)	0.086 (2)	0.0437 (17)	0.0276 (17)	0.0025 (14)	0.0096 (16)
Cl2	0.0496 (17)	0.0452 (16)	0.0528 (18)	−0.0137 (13)	−0.0019 (14)	−0.0005 (13)
N1	0.056 (6)	0.044 (5)	0.031 (5)	0.020 (5)	0.023 (5)	0.012 (4)
N2	0.034 (5)	0.045 (5)	0.033 (5)	0.012 (4)	0.011 (4)	0.014 (4)
N3	0.032 (5)	0.053 (6)	0.030 (5)	−0.005 (4)	0.009 (4)	0.000 (4)
N4	0.036 (5)	0.057 (6)	0.035 (5)	−0.011 (5)	0.000 (4)	−0.008 (5)
C1	0.053 (7)	0.062 (8)	0.041 (7)	0.012 (6)	0.021 (6)	0.011 (6)
C2	0.079 (10)	0.037 (7)	0.051 (7)	0.012 (6)	0.033 (7)	0.006 (6)
C3	0.083 (10)	0.040 (7)	0.057 (8)	0.018 (6)	0.035 (8)	0.015 (6)
C4	0.073 (9)	0.035 (6)	0.036 (6)	0.014 (6)	0.021 (6)	0.000 (5)
C5	0.047 (7)	0.045 (6)	0.029 (6)	0.019 (5)	0.013 (5)	0.006 (5)
C6	0.051 (7)	0.033 (6)	0.033 (6)	0.005 (5)	0.023 (5)	0.006 (5)
C7	0.049 (7)	0.036 (6)	0.033 (6)	0.011 (5)	0.021 (5)	0.008 (5)
C8	0.038 (6)	0.024 (5)	0.038 (6)	−0.003 (4)	0.011 (5)	−0.002 (4)
C9	0.038 (6)	0.031 (5)	0.027 (5)	0.007 (4)	0.010 (5)	0.006 (4)
C10	0.039 (6)	0.031 (5)	0.032 (5)	0.006 (4)	0.006 (5)	0.007 (4)
C11	0.018 (5)	0.045 (6)	0.034 (6)	0.007 (4)	0.005 (4)	0.006 (5)
C12	0.029 (6)	0.065 (8)	0.035 (6)	0.001 (5)	0.009 (5)	0.001 (6)
C13	0.029 (6)	0.074 (9)	0.043 (7)	−0.009 (6)	0.000 (5)	−0.011 (6)
C14	0.041 (7)	0.052 (7)	0.038 (6)	−0.014 (5)	0.013 (6)	−0.010 (5)
C15	0.026 (5)	0.058 (7)	0.034 (6)	0.002 (5)	0.012 (5)	−0.001 (5)
C16	0.051 (7)	0.031 (5)	0.031 (5)	−0.003 (5)	0.021 (5)	−0.006 (4)
C17	0.046 (7)	0.034 (5)	0.031 (5)	−0.006 (5)	0.008 (5)	−0.007 (5)
C18	0.032 (6)	0.036 (6)	0.036 (6)	0.000 (5)	−0.001 (5)	−0.002 (5)
C19	0.042 (6)	0.049 (7)	0.026 (5)	0.002 (5)	0.010 (5)	−0.017 (5)
C20	0.036 (6)	0.031 (5)	0.030 (5)	−0.016 (4)	0.005 (5)	−0.006 (4)
C21	0.040 (6)	0.034 (6)	0.034 (6)	−0.008 (4)	0.014 (5)	0.000 (4)

### Geometric parameters (Å, °)

**Table:**

Pd—N1	2.034 (10)	C7—H7	0.9500
Pd—N2	1.922 (9)	C8—C9	1.414 (14)
Pd—N3	2.022 (10)	C8—C16	1.451 (16)
Pd—Cl1	2.290 (3)	C9—C10	1.376 (15)
N1—C1	1.357 (15)	C9—H9	0.9500
N1—C5	1.359 (15)	C10—C11	1.466 (15)
N2—C6	1.333 (14)	C11—C15	1.395 (15)
N2—C10	1.357 (13)	C12—C13	1.379 (17)
N3—C12	1.312 (14)	C12—H12	0.9500
N3—C11	1.375 (13)	C13—C14	1.378 (17)
N4—C19	1.377 (14)	C13—H13	0.9500
N4—H1N	0.8800 (11)	C14—C15	1.403 (15)
N4—H2N	0.8800 (10)	C14—H14	0.9500
C1—C2	1.382 (19)	C15—H15	0.9500
C1—H1	0.9500	C16—C21	1.414 (14)
C2—C3	1.37 (2)	C16—C17	1.426 (15)
C2—H2	0.9500	C17—C18	1.387 (15)
C3—C4	1.394 (17)	C17—H17	0.9500
C3—H3	0.9500	C18—C19	1.401 (15)
C4—C5	1.374 (17)	C18—H18	0.9500
C4—H4	0.9500	C19—C20	1.370 (15)
C5—C6	1.492 (15)	C20—C21	1.377 (15)
C6—C7	1.371 (16)	C20—H20	0.9500
C7—C8	1.404 (15)	C21—H21	0.9500
			
N2—Pd—N3	81.3 (4)	C9—C8—C16	121.2 (9)
N2—Pd—N1	80.3 (4)	C10—C9—C8	120.8 (9)
N3—Pd—N1	161.6 (4)	C10—C9—H9	119.6
N2—Pd—Cl1	179.2 (3)	C8—C9—H9	119.6
N3—Pd—Cl1	98.8 (3)	N2—C10—C9	119.0 (10)
N1—Pd—Cl1	99.6 (3)	N2—C10—C11	112.7 (9)
C1—N1—C5	119.3 (11)	C9—C10—C11	128.3 (9)
C1—N1—Pd	126.7 (9)	N3—C11—C15	120.8 (10)
C5—N1—Pd	114.0 (7)	N3—C11—C10	115.0 (9)
C6—N2—C10	122.6 (9)	C15—C11—C10	124.1 (9)
C6—N2—Pd	119.3 (7)	N3—C12—C13	123.8 (11)
C10—N2—Pd	118.1 (8)	N3—C12—H12	118.1
C12—N3—C11	118.6 (10)	C13—C12—H12	118.1
C12—N3—Pd	128.7 (8)	C14—C13—C12	119.0 (11)
C11—N3—Pd	112.7 (7)	C14—C13—H13	120.5
C19—N4—H1N	121 (7)	C12—C13—H13	120.5
C19—N4—H2N	121 (7)	C13—C14—C15	118.6 (12)
H1N—N4—H2N	109 (8)	C13—C14—H14	120.7
N1—C1—C2	120.3 (13)	C15—C14—H14	120.7
N1—C1—H1	119.8	C11—C15—C14	119.1 (10)
C2—C1—H1	119.8	C11—C15—H15	120.5
C3—C2—C1	121.1 (12)	C14—C15—H15	120.5
C3—C2—H2	119.5	C21—C16—C17	115.1 (10)
C1—C2—H2	119.5	C21—C16—C8	122.2 (9)
C2—C3—C4	118.0 (13)	C17—C16—C8	122.6 (9)
C2—C3—H3	121.0	C18—C17—C16	121.3 (10)
C4—C3—H3	121.0	C18—C17—H17	119.3
C5—C4—C3	119.7 (13)	C16—C17—H17	119.3
C5—C4—H4	120.2	C17—C18—C19	121.5 (10)
C3—C4—H4	120.2	C17—C18—H18	119.3
N1—C5—C4	121.6 (10)	C19—C18—H18	119.3
N1—C5—C6	113.5 (10)	C20—C19—N4	121.9 (10)
C4—C5—C6	124.8 (11)	C20—C19—C18	117.6 (10)
N2—C6—C7	120.1 (10)	N4—C19—C18	120.4 (10)
N2—C6—C5	112.8 (10)	C19—C20—C21	122.0 (9)
C7—C6—C5	127.1 (11)	C19—C20—H20	119.0
C6—C7—C8	120.7 (11)	C21—C20—H20	119.0
C6—C7—H7	119.6	C20—C21—C16	122.4 (10)
C8—C7—H7	119.6	C20—C21—H21	118.8
C7—C8—C9	116.8 (10)	C16—C21—H21	118.8
C7—C8—C16	122.0 (9)		
			
N2—Pd—N1—C1	−176.1 (9)	C6—C7—C8—C16	179.9 (9)
N3—Pd—N1—C1	−173.8 (9)	C7—C8—C9—C10	2.2 (14)
Cl1—Pd—N1—C1	3.2 (9)	C16—C8—C9—C10	−179.6 (9)
N2—Pd—N1—C5	1.9 (7)	C6—N2—C10—C9	−0.7 (15)
N3—Pd—N1—C5	4.2 (15)	Pd—N2—C10—C9	177.8 (7)
Cl1—Pd—N1—C5	−178.8 (6)	C6—N2—C10—C11	179.7 (9)
N3—Pd—N2—C6	178.0 (8)	Pd—N2—C10—C11	−1.8 (11)
N1—Pd—N2—C6	−2.7 (7)	C8—C9—C10—N2	−0.9 (15)
N3—Pd—N2—C10	−0.5 (7)	C8—C9—C10—C11	178.6 (10)
N1—Pd—N2—C10	178.7 (8)	C12—N3—C11—C15	−1.7 (14)
N2—Pd—N3—C12	−176.4 (9)	Pd—N3—C11—C15	179.0 (7)
N1—Pd—N3—C12	−178.7 (9)	C12—N3—C11—C10	174.7 (9)
Cl1—Pd—N3—C12	4.4 (9)	Pd—N3—C11—C10	−4.6 (11)
N2—Pd—N3—C11	2.9 (7)	N2—C10—C11—N3	4.2 (13)
N1—Pd—N3—C11	0.6 (14)	C9—C10—C11—N3	−175.3 (9)
Cl1—Pd—N3—C11	−176.3 (6)	N2—C10—C11—C15	−179.5 (9)
C5—N1—C1—C2	−0.5 (15)	C9—C10—C11—C15	0.9 (17)
Pd—N1—C1—C2	177.4 (8)	C11—N3—C12—C13	0.4 (16)
N1—C1—C2—C3	1.9 (17)	Pd—N3—C12—C13	179.7 (8)
C1—C2—C3—C4	−2.3 (17)	N3—C12—C13—C14	1.4 (18)
C2—C3—C4—C5	1.3 (17)	C12—C13—C14—C15	−1.9 (17)
C1—N1—C5—C4	−0.5 (15)	N3—C11—C15—C14	1.1 (15)
Pd—N1—C5—C4	−178.7 (8)	C10—C11—C15—C14	−174.9 (9)
C1—N1—C5—C6	177.1 (9)	C13—C14—C15—C11	0.7 (15)
Pd—N1—C5—C6	−1.0 (11)	C7—C8—C16—C21	−10.2 (15)
C3—C4—C5—N1	0.1 (16)	C9—C8—C16—C21	171.7 (9)
C3—C4—C5—C6	−177.3 (10)	C7—C8—C16—C17	171.5 (9)
C10—N2—C6—C7	0.9 (15)	C9—C8—C16—C17	−6.6 (15)
Pd—N2—C6—C7	−177.6 (7)	C21—C16—C17—C18	3.5 (14)
C10—N2—C6—C5	−178.7 (9)	C8—C16—C17—C18	−178.1 (9)
Pd—N2—C6—C5	2.9 (11)	C16—C17—C18—C19	−0.8 (16)
N1—C5—C6—N2	−1.0 (12)	C17—C18—C19—C20	−2.4 (15)
C4—C5—C6—N2	176.5 (10)	C17—C18—C19—N4	−179.9 (9)
N1—C5—C6—C7	179.4 (9)	N4—C19—C20—C21	−179.8 (9)
C4—C5—C6—C7	−3.0 (17)	C18—C19—C20—C21	2.8 (15)
N2—C6—C7—C8	0.5 (15)	C19—C20—C21—C16	0.1 (16)
C5—C6—C7—C8	180.0 (9)	C17—C16—C21—C20	−3.2 (14)
C6—C7—C8—C9	−1.9 (14)	C8—C16—C21—C20	178.4 (9)

### Hydrogen-bond geometry (Å, °)

**Table:**

*D*—H···*A*	*D*—H	H···*A*	*D*···*A*	*D*—H···*A*
N4—H1n···Cl2	0.88 (9)	2.42 (7)	3.287 (11)	167 (8)
N4—H2n···Cl2^i^	0.88 (7)	2.48 (8)	3.352 (10)	173 (10)
C13—H13···Cl1^ii^	0.95	2.61	3.493 (14)	156
C15—H15···Cl2^iii^	0.95	2.56	3.449 (12)	155

Symmetry codes: (i) −*x*+3, −*y*+1, −*z*+1; (ii) −*x*+1/2, *y*−1/2, −*z*−1/2; (iii) −*x*+5/2, *y*−1/2, −*z*+1/2.
